# Influence of Vitamin D Deficiency on Inflammatory Markers and Clinical Disease Activity in IBD Patients

**DOI:** 10.3390/nu11051059

**Published:** 2019-05-11

**Authors:** Pedro López-Muñoz, Belén Beltrán, Esteban Sáez-González, Amparo Alba, Pilar Nos, Marisa Iborra

**Affiliations:** 1 IBD Unit, Department of Gastroenterology, Hospital Universitari i Politècnic la Fe, 46026 Valencia, Spain; pedro.lopez8928@gmail.com (P.L.-M.); esteban.digestivo@gmail.com (E.S.-G.); nos_pil@gva.es (P.N.); marisaiborra@hotmail.com (M.I.); 2 IBD Research Group, Medical Research Institute Hospital la Fe (IIS La Fe), 46026 Valencia, Spain; 3 Networked Biomedical Research Center for Hepatic and Digestive Diseases (CIBEREHD), Institute of Health Carlos III, 28029 Madrid, Spain; 4 Department of Clinical Analysis, Hospital Universitari i Politècnic la Fe, 46026 Valencia, Spain; alba_amp@gva.es

**Keywords:** vitamin D, Crohn’s disease, ulcerative colitis, faecal calprotectin, C-reactive protein

## Abstract

Vitamin D has recently been discovered to be a potential immune modulator. Low serum vitamin D levels have been associated with risk of relapse and exacerbation of clinical outcomes in Crohn’s disease (CD) and ulcerative colitis (UC). A retrospective, longitudinal study was conducted to determine the association between vitamin D levels and inflammatory markers and clinical disease activity in inflammatory bowel disease (IBD). In addition, circulating 25(OH)D_3_ progression was evaluated according to vitamin D supplementation. Participants were separated into three groups according to their vitamin D level: severe deficiency (SD), moderate deficiency (MD) and sufficiency (S). Serum 25(OH)D_3_ was inversely correlated with faecal calprotectin (FC) for CD and UC but was only correlated with C-reactive protein (CRP) for UC patients. In the multivariate analysis of FC, CRP and fibrinogen (FBG), we predicted the presence of a patient in the SD group with 80% accuracy. A deficiency of 25(OH)D_3_ was associated with increased hospitalisations, flare-ups, the use of steroids and escalating treatment. Supplemental doses of vitamin D were likely to be insufficient to reach adequate serum levels of 25(OH)D_3_. Vitamin D intervention studies are warranted to determine whether giving higher doses of vitamin D in IBD might reduce intestinal inflammation or disease activity.

## 1. Introduction

Vitamin D is a lipophilic hormone synthesised in the skin under the influence of ultraviolet (UV) sunlight. Although most foods contain little vitamin D, it can be obtained from the diet to a lesser extent. The most important sources of this vitamin are fatty fish and egg yolk [1,2].

**Figure 1:**
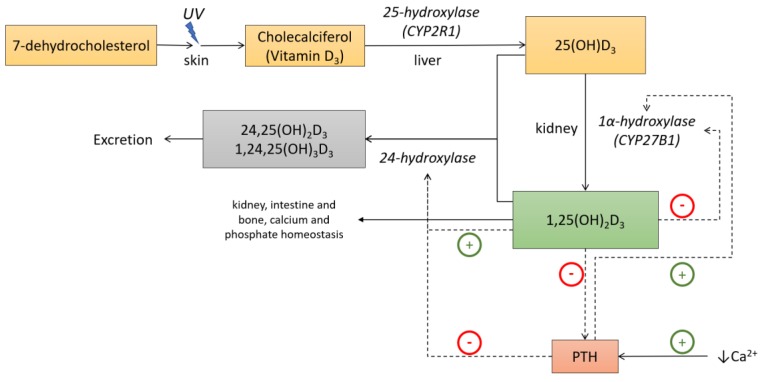
Vitamin D metabolism and functions. Under ultraviolet B light (UVB) exposure, 7-dehydroxycholesterol is converted to vitamin D_3_ in the skin. First, hydroxylation occurs in the liver where it is converted to 25-hydroxyvitamin D_3_. 25-hydroxyvitamin D_3_ is further converted in the kidney to its active metabolite, 1α,25-dihydroxyvitamin D_3_. In the kidney, 1α-hydroxylase is stimulated by the parathyroid hormone (PTH) and feedback inhibited by 1α,25-dihydroxyvitamin D_3_. 1α,25-dihydroxyvitamin D_3_ targets the intestine, kidney and bone to regulate calcium and phosphate homeostasis. Adapted from Lim et al [6].

UV type B light exposure causes 7-dehydrocholesterol transformation into cholecalciferol or vitamin D_3_. However, this UV-mediated conversion varies with time of the year, latitude and altitude. During approximately six months of the year, UVB radiation intensity is insufficient for vitamin D synthesis at 45° latitude near sea level (e.g., France or Italy). This period of time increases with the distance from the equator and is also known as vitamin D winter [3]. Along these lines, in countries where there is limited skin exposure to sunlight a dietary supply of vitamin D is important. Cholecalciferol is hydroxylated in the liver into 25-hydroxyvitamin D3 (25(OH)D3) and subsequently in the kidney into 1,25-dihydroxyvitamin D3 (1,25(OH)2D3), or calcitriol, the active metabolite [4] (Figure 1). These two steps are catalysed by the enzymes 25-hydroxylase (CYP2R1) and 1α-hydroxylase (CYP27B1) that belong to the family of cytochrome P450 mixed function oxidases (CYPs). CYP2R1 has only been identified in the microsomal fraction of liver. However, although the kidney is the main source of 1,25(OH)_2_ D_3_, several other tissues also express CYP27B1. The regulation of the renal CYP27B1 differs from that of the extrarenal version [5]. In this way, renal CYP27B1 is activated by the parathyroid hormone (PTH) and inhibited by fibroblast growth factor 23 (FGF23) as well as by 1,25(OH)_2_D_3_ itself. Elevated calcium suppresses CYP27B1 by means of the suppression of PTH, and elevated phosphate suppresses CYP27B1 by means of FGF23 stimulation, although these ions can have direct effects on renal CYP27B1 on their own [5]. Thus, it is known that CYP27B1 is expressed in numerous tissues where vitamin D_3_ can act as an intracrine or paracrine signal. In this way, several immune system cell types express both CYP27B1 and the vitamin D receptor, with CYP27B1 production controlled by a number of immune-specific inputs [3].

The half-life of 1,25(OH)_2_D_3_ is very short, ranging from 20 to 40 hours [7]; therefore, its serum determination is not clinically relevant. However, the 25(OH)D_3_ half-life ranges from 12 to 19 days [8], so it appears to be the most reliable source of systemic vitamin D. 1,25(OH)_2_D_3_ has its effect on the classic target organs such as bone, intestine and kidney and stimulates calcium transport from these organs to the peripheral blood stream [4].

Calcitriol can influence the expression of targets genes by binding to the vitamin D receptor (VDR), which is present in many cell populations and tissues, such as immune cells. Vitamin D promotes the intestinal absorption of calcium and phosphate and is critical for bone growth and remodelling. Now, however, it is well established that the physiological importance of vitamin D levels extends beyond its classical role in calcium and bone metabolism. Serum vitamin D levels have been associated with inflammatory diseases, such as inflammatory bowel disease (IBD), rheumatoid arthritis, systemic lupus erythematosus, multiple sclerosis, atherosclerosis, and asthma. Regarding inflammation, it has become clear that vitamin D inhibits production of proinflammatory cytokines like IL-6 or TNFα in monocytes via the inhibition of p38 MAP kinase [9], and it can promote anti-inflammatory T-cell pathways to stimulate the antimicrobial effects of macrophages [10].

In terms of gut homeostasis, vitamin D plays a role in protecting the intestinal epithelial barrier, immunity and microbiota, all of which are directly involved in IBD [11].

In-vitro 1,25(OH)2D3 treatment can protect the intestinal epithelial barrier in ulcerative colitis (UC) patients by regulating various pathways involved in tight junction proteins [12]

Similar studies have proposed that lack of VDR leads to hyperfunction of Claudin-2. Claudin-2 is an epithelial protein that forms a water channel to permit the paracellular passage of water through its pores in the epithelium, and its expression is restricted to colonic proliferative cells. An overexpression of Claudin-2 permits intestinal leakage, and is associated with active disease in IBD and reduction of intestinal VDR [13].

Other genetic changes in the VDR gene can contribute to a greater risk for CD. Patients with Crohn’s disease (CD) who are homozygous for a certain single nucleotide polymorphism (SNP) in the VDR gene have lower levels of VDR protein expressed in serum monocytes. These individuals have an overactivation of lymphocytic adhesion molecules and a higher risk of developing a penetrating phenotype of CD [14].

Vitamin D has effects on both innate and adaptive immune pathways. 1,25(OH)2D3 modulates the expression of cationic antimicrobial peptides like defensins and cathelicidins. VDR signals through distal enhancers in the NOD2 gene, leading to the expression of defensin beta 2 in macrophages. However, this response was absent in macrophages from patients with CD homozygous for nonfunctional NOD2 variants [15]. It should be noted that NOD2 is also known as IBD1. This gene is located on chromosome 16, in the susceptibility locus for CD, which is responsible for familial aggregation of this disease [16]. It has also been reported that DNA copy number of the beta-defensin gene cluster is highly polymorphic within the healthy population, whereas CD patients appear to present a lower copy number. This results in a diminished beta-defensin expression, predisposing patients to major susceptibility to colonic CD [17]. An induction of human cathelicidin antimicrobial peptide gene was observed under the influence of 1,25(OH)2D3 in colon cancer cells and other tissues [18,19]. Vitamin D is also involved in immune regulation by modulating the role of dendritic cells and macrophages through two methods: firstly, 1,25(OH)2D3 inhibits IL-12, a pivotal interleukin in Th1 development [20]; secondly, it promotes IL-10 to stop maturation from dendritic cells to serum monocytes and leads to apoptosis in mature dendritic cells [21]. Regarding adaptive immunity, 1,25(OH)2D3 affects T-cell polarisation by inhibiting T helper 1(Th) (IFN-gamma production) and promoting Th2 cell development (IL-4, IL-5, and IL-10 production) from naïve T-cells [22]. It has been demonstrated in controlled studies that higher vitamin D levels in UC patients were associated with protective anti-inflammatory serum cytokine profiles [23].

Gut microbiota have become the main research topic in intestinal homeostasis. One recent study demonstrated that individuals with significant intake of vitamin D had different microbiota strains from those without a significant vitamin D intake. These patients had plenty of Bacteroidetes, Prevotella and Megasphera strains, traditionally associated with a noninflammatory status [24]. In another study, administration of vitamin D to CD patients resulted in significant change in the gut microbiota with a high abundance of some strains such as *Alistipes* and *Roseburia* that were not observed in healthy controls [25]. In murine induced colitis, *Alistipes finegoldii* played a protective role [26]. *Roseburia* genii are part of commensal bacteria that produce short-chain fatty acids, especially butyrate, affecting colonic motility, immunity maintenance and anti-inflammatory properties [27]. Therefore, administration of vitamin D might have a positive effect on CD by modulating the intestinal bacterial composition and also by increasing the abundance of potential beneficial bacterial strains [25].

Regarding the epidemiology of vitamin D deficiency in IBD, there is a systematic review and meta-analysis that defined this deficiency as serum 25(OH)D3 below 25 ng/mL. In this study, the prevalence was 38.1% in CD and 31.6% in UC [28].

The latest evidence suggests that low vitamin D levels are involved in changing disease activity and inflammatory markers in IBD. However, these studies sometimes fail to confirm which comes first: are vitamin D levels an independent predictor of clinical activity or it is just a mere bystander of increased inflammation?

Due to chronic malabsorption, IBD patients and particularly those with CD are at greater risk of certain nutritional deficiencies. Micronutrients, including hydro and lipophilic vitamins, iron, calcium and zinc, are the most common problems [29]. Malabsorption, maldigestion and upper protein requirements are directly related to clinical disease activity.

It is unknown why quiescent IBD has a greater prevalence of vitamin D deficiency than other risk groups [30]. Even in patients with IBD in clinical remission, vitamin D malabsorption has been confirmed. This fact led to controversy among researchers about oral intake as standard supplementation. According to several prospective studies, IBD patients with vitamin D deficiency are at greater risk of relapse, hospitalisations and flare-ups [2,31,32,33,34,35]. Schäffler et al. described a correlation between the use of a TNF-alpha inhibitor and higher vitamin D levels in CD patients. This correlation could be explained by a better disease control of these patients [34]. However, they detected a correlation between a higher disease activity and lower vitamin D levels in UC but not in CD. Moreover, the same authors found that in CD located in the small intestine, CD patients after small intestine resections showed significant lower vitamin D levels. Other resections did not lead to changes in the vitamin D levels; therefore, the small intestine plays an important role in vitamin D absorption, particularly in IBD [34]. Despite the immune-modulation role of vitamin D confirmed in translational studies, knowledge about its molecular action in IBD is still scarce. Exhaustive study of vitamin D immune pathways is imperative to identifying new supplementation strategies that correct this deficiency. It is also necessary to establish true sufficiency levels of vitamin D to have a goal for supplementation therapy.

As a result of these findings, our group posed a study to assess outcomes in IBD activity depending on vitamin D status. Thus, the association between vitamin D deficiency and inflammatory markers and clinical disease activity could be evaluated. We also hypothesised that oral supplementation according to practice guidelines was not enough to acquire vitamin D sufficiency.

## 2. Materials and Methods 

### 2.1. Study Design and Patient Enrolment

We conducted a retrospective, longitudinal and observational study of patients with UC and CD, defined by European Crohn’s and Colitis Organization (ECCO) guidelines criteria, which had serial determinations of vitamin D serum samples from the IBD Unit of Hospital Universitari i Politècnic la Fe, a tertiary centre, between 2015 and 2018. Formal consent was collected from all the patients and an ethical committee, according to the declaration of Helsinki, approved the study.

**Figure 2:**
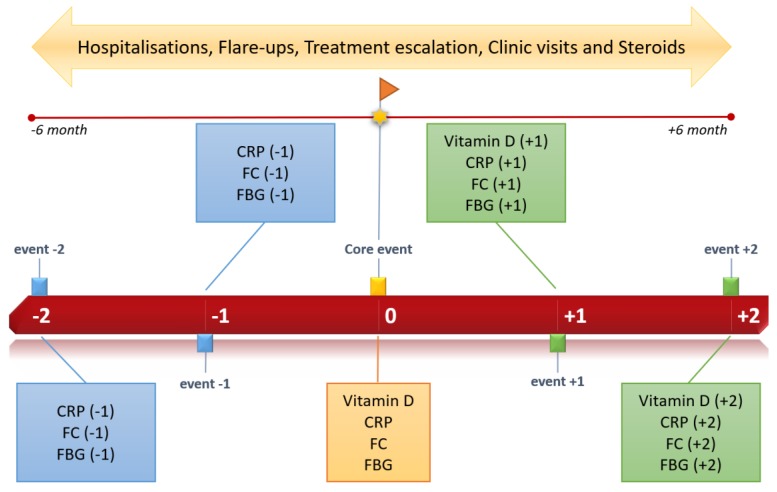
Study timeline.

A total of 84 patients divided into three groups according to the 25(OH)D3 (ng/mL) level were enrolled in our study. The first vitamin D serum sample collected was used as a baseline to separate patients into groups and according to core event. A cut-off of <15 ng/mL was considered severe deficiency (SD), >15 and <30 ng/mL moderate deficiency (MD) and >30 ng/mL sufficiency (S). Progression of vitamin D and supplementation from baseline was evaluated. Clinical disease activity (flare-ups, hospitalisations, times to visit IBD clinic, escalating treatment, use of steroids) in the six months before and the six months after the core event was monitored. In addition, inflammatory markers (CRP, FC and fibrinogen (FBG)) were collected to explore the inflammatory status around the vitamin D core event. Two determinations before and two determinations after to the core event were needed for patient inclusion (Figure 2). Two or more consecutive 25(OH)D3 determinations were also required. Other relevant descriptive variables: age, sex, IBD (EC or UC), extraintestinal manifestations, and current therapy (anti-TNF, immunomodulators, etc.) were considered. We sought active supplementation, establishing at least 25,000 IU (international units) of oral, subcutaneous or intramuscular cholecalciferol monthly as adequate supplementation.

Patients under 18 years, pregnant patients, those with a previous colectomy in UC, those who had CD surgery in the last three months, those with colorectal cancer or another tumour, those with a severe infection during the time of study and those with malabsorption disease were excluded from enrolment.

Disease activity and laboratory markers were collected from our software OrionClinic (Version 11). Suitable patients to enroll in this study were found by crossing vitamin D measurements from iGestlab software (Cointec iGestlab © 2013. v1.9.9.815, Orihuela, Alicante, Spain) and IBD Unit clinical histories. Patients with several vitamin D samples, inflammatory markers and IBD diagnosis were filtered to assess enrolment.

Serum 25(OH)D_3_ levels (ng/mL) were measured by automated chemiluminescence analyser LIAISON^®^ XL (DiaSorin, Sialuggia, Italy).

A total of 252 patients from IBD Unit and vitamin D measurements were evaluated to enroll. Ultimately, 41 patients with SD, 28 with MD and 16 with sufficient vitamin D levels met the inclusion criteria.

### 2.2. Statistical Analysis

An ANOVA test on ranks (Kruskal–Wallis test) was performed to compare inflammatory markers with the three different Vitamin D groups (severe deficiency, moderate deficiency and sufficiency). Moreover, a multiple comparison procedure was conducted to quantify the extent of the differences between the groups. The Pearson correlation test was applied to study calprotectin-CRP-fibrinogen correlations with vitamin D. The evolution of vitamin D, FC, CRP and FBG was evaluated by mixed linear regression models. A random effect factor was included to amend the nonindependence of the data (longitudinal study).

Given some patients were supplemented with vitamin D, a comparative study was done with this group and the nonsupplemented ones to analyse the evolution of this parameter. Additionally, the association between vitamin D levels and markers of clinical activity (hospitalisations, flare-ups, escalating treatment, number of times they visited the clinic and the use of steroids) was studied through logistic regression models.

All models included the covariates sex, age and type of disease (CD or UC) as possible confounding factors. R statistic software (3.5.1 version, https://www.r-project.org/), Sigmaplot (SYSTAT, San José, CA, USA) and Minitab (State College, PA, USA) were used in this work. A significance level of 5% was selected. Multivariate discriminant analysis was performed with SIMCA-P (Umetrics, Umeå, Sweden).

## 3. Results

This study evaluated three concerns: Firstly, the correlation between vitamin D levels, expressed as 25(OH)D3, with both systemic and intestinal inflammation in IBD patients. Secondly, the association between vitamin D deficiency and clinical disease activity markers, collected in a period of time between six months before and after the randomly selected vitamin D core event. During this period, data on admissions, flare-ups, times the patient needed to visit the clinic, the use of steroids and the need for escalating treatment were collected. Thirdly, which patients benefitted from active supplementation was explored. 

### 3.1. Descriptive Statistics

**Figure 3:**
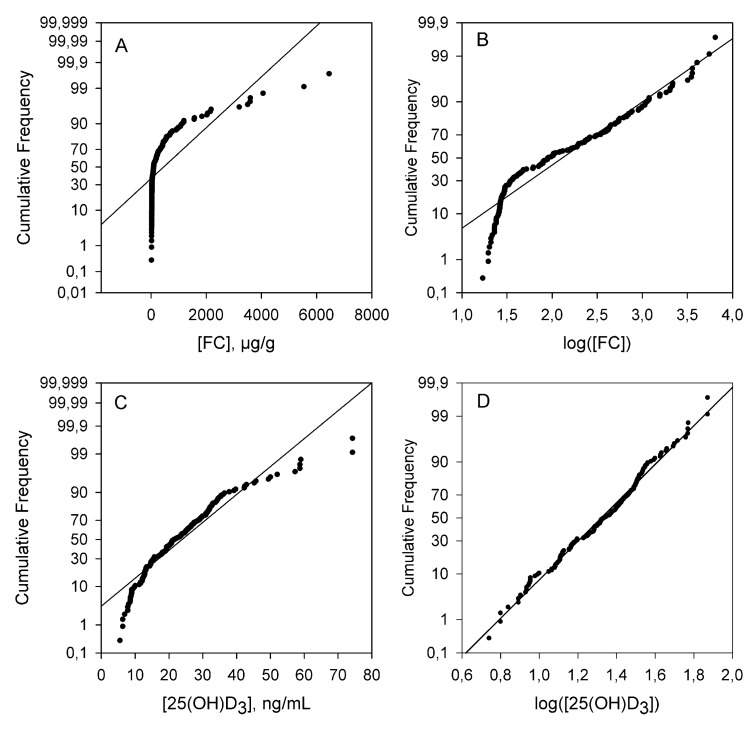
Normality study of FC (**A**,**B**) and vitamin D (**C**,**D**). Note the nonparametric distribution of FC.

First, a normality study for the continuous quantitative variables (vitamin D, FC, CRP and FBG) was carried out. None of the inflammatory markers (FC, CRP, FBG) followed a normal distribution but their logarithmic transformation leads to data that were normally distributed (Figure 3). However, the vitamin D central values did follow a normal distribution. 

Table 1 and Table 2 show some of the statistical parameters obtained from the descriptive parametric and nonparametric study. The data have been grouped according to their vitamin D content. The central and dispersion values derived from the parametric and nonparametric treatment of the data are shown. Table 1 shows the parametric descriptive statistics where the deviation of the data with respect to normal behavior is clearly shown. The high values of MSSD (mean of the squared successive differences) clearly indicate a non-random distribution of the results. To obtain a more accurate description of central values and dispersion of inflammatory markers according to their vitamin D content, a robust descriptive statistical study is shown in Table 2. The median of the of inflammatory markers tends to increase when lower vitamin D levels were found. The significant differences found between the groups are noteworthy. The median of all determinations of FC in the group SD was 345.7 μg/g, while in the MD and S groups it was 63.5 and 29.7 μg/g, respectively. The medians of the CRP determinations in the SD, MD and S groups were 5.6, 1.5 and 1.4 mg/dL, respectively.

### 3.2. Qualitative Variables Study

Other qualitative variables were also considered and appear in Table 3. The mean age of the patients was 43.7 years, and 39 of the patients were women (46%). Some 60 patients had CD (71%) and 25 presented as UC (29%). Forty-four patients (52%) were supplemented with vitamin D, almost all of them in the SD group. Extraintestinal manifestations were clustered into articular (eight patients) and dermatological manifestations (two patients). Four cases of CD with perianal disease were observed. We collected data on the treatments given during the period of study: biologics (anti-TNFα, vedolizumab, ustekinumab), immunosuppressive agents (azathioprine, methotrexate, 6-mercaptopurine and tacrolimus) and the need for steroids therapy.

Six patients from the SD group required a combination of vedolizumab and steroids during the time of study. None of the individuals in the MD or S groups were on vedolizumab. Some 30 patients from the SD group were on anti-TNFα therapy, 12 of them in combination with azathioprine and eight of them with steroids requirement. However, only 17 patients were on anti-TNFα therapy in MD and S groups altogether. Only three of them required steroids and seven patients were on combination therapy with azathioprine.

**Figure 4:**
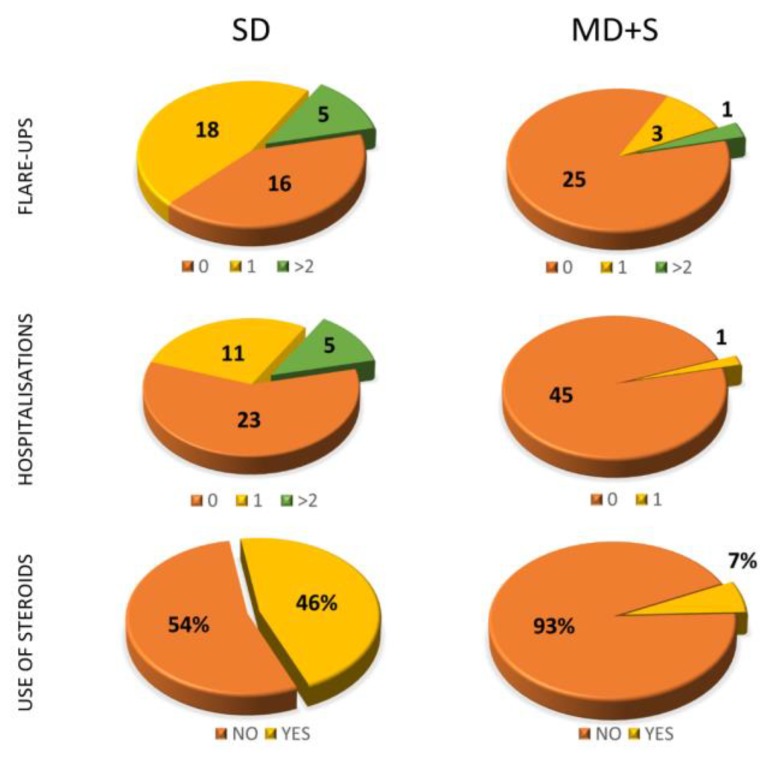
Results of the study of certain qualitative variables depending on vitamin D levels (SD or MD+S).

Clinical disease activity markers collected included flare-ups, hospitalisations, number of times patients visited the clinic, changing or escalating treatment and need for steroid therapy. We found remarkable results between the groups according to vitamin D levels. Among the patients with SD, 16 patients (41%) were hospitalised at least once and 23 patients (58.9%) had at least one flare-up during the time of the study (Figure 4). In the MD group along with the S group, only four flare-ups and one hospitalisation were reported. In the SD group, patients visited the clinic an average of 5.33 times, while in the MD and S groups they visited 3.61 times.

Changes or escalations in current therapy were made for 69.23% of the patients with SD, while only 8.7% in the MD and S groups required such changes. Eighteen patients in the SD group (46.15%) required steroids during the time of study, while only three patients in the SD and S groups did (6.53%).

### 3.3. Linear Correlation Study of Quantitative Variables

Table 4 shows the median and interquartile range values of inflammatory markers. In nonsupplemented patients, the median and interquartile range of FC increased in cases of severe deficiency. This also occurred when vitamin D was supplemented, but to a lesser extent. A similar behaviour was observed for CRP in terms of the median but not for the interquartile range.

**Figure 5:**
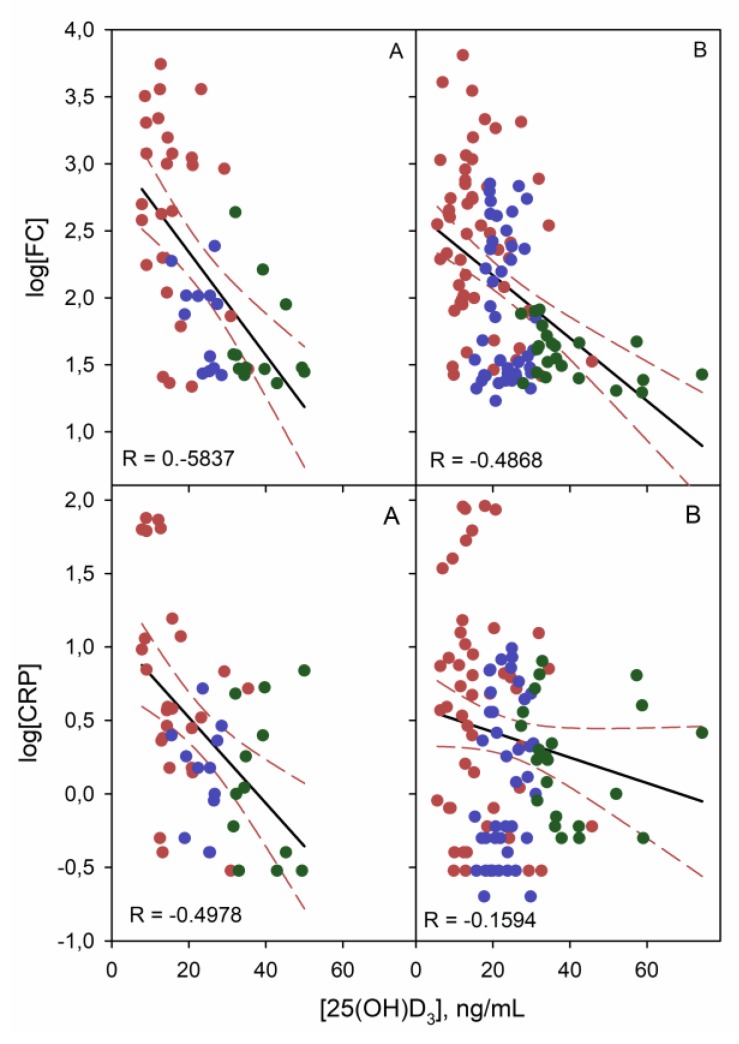
Linear correlation graphs of FC and CRP (log transform) with vitamin D for UC (**A**) and CD (**B**) patients. Red, blue and green points correspond to SD, MD and S vitamin D level, respectively.

A nonparametric linear correlation study of vitamin D with FC, CRP and FBG was performed. A Pearson correlation coefficient (R) of −0.533 (*p* < 0.001) was obtained for vitamin D and FC in all patients. When two groups of patients were considered, both CD and UC showed a good correlation (Figure 5). As can be seen, this correlation was almost independent of the disease, given there was no significant differences between both slopes for UC and CD (*p* = 0.686). 

For CRP, in patients with UC and CD, a Pearson correlation coefficient of −0.512 and −0.16 (*p* = 0.01 and 0.256), respectively, was obtained (Figure 5). These results demonstrate a strong correlation between CRP and vitamin D exclusively in patients with UC, not in CD. Therefore, there are significant differences between the correlation slopes (*p* = 0.02). No linear correlation was found between FBG values and vitamin D levels (R near 0).

**Figure 6:**
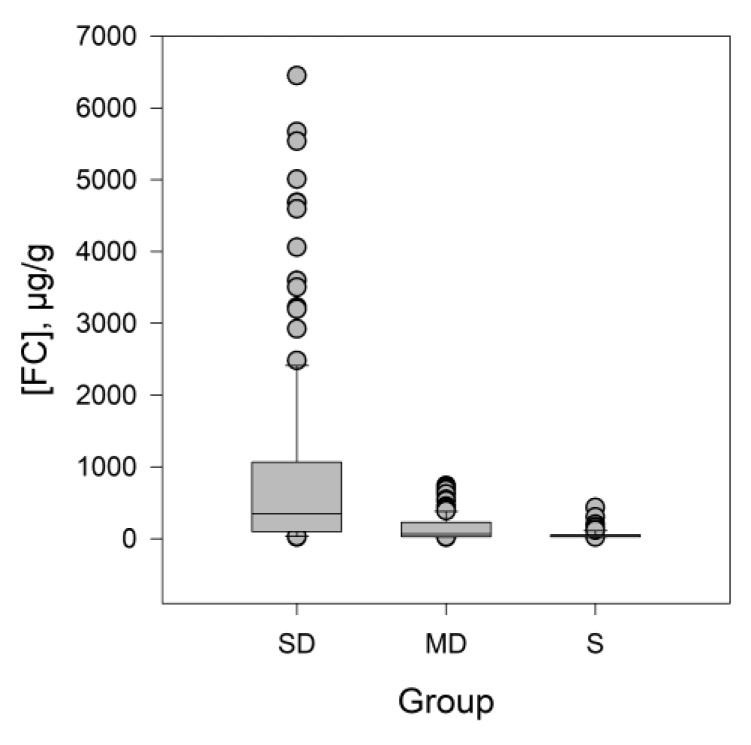
Box and whisker plot to represent the nonparametric variable FC, the values inside the box conform the 25th and 75th percentile, while the horizontal line that crosses the box marks the median. The values outside the whiskers are considered atypical.

**Figure 7:**
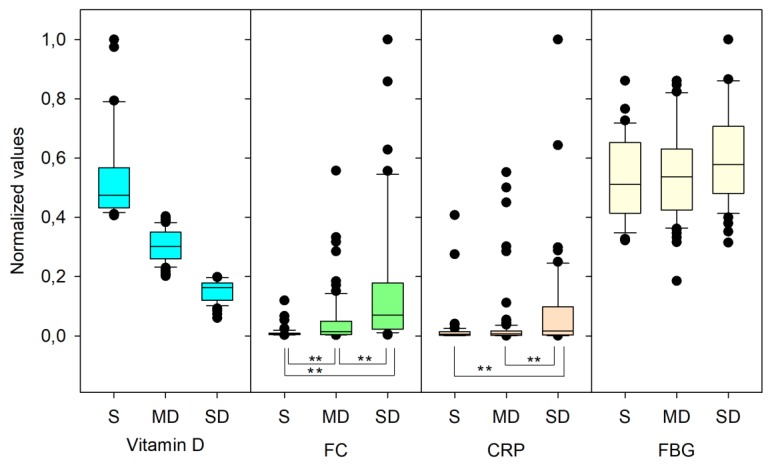
Box and whisker plot using normalised values of inflammatory markers depending on vitamin D levels. (**) correspond to significant differences at 99% (Dun’s test for multiple comparisons on the Kruskal–Wallis test).

The statistical normality study of FC, CRP and FBG showed a non-normal distribution and, as a consequence, a Kruskal–Wallis test for groups comparison was performed. Significant differences for FC were found between the medians of the SD (345 µg/g), MD (46 µg/g) and S (30 µg/g) groups with a *p*-value < 0.001 (Figure 6). A similar study for CRP also showed significant differences (*p* = 0.01) while no differences were obtained for FBG. Figure 7 shows box and whisker plots of inflammatory markers depending on vitamin D levels. Data were normalised for representation purposes. As can be seen, there were significant differences at 99% (**) in the contents of CRP and FC depending on the vitamin D levels. 

A multivariate discriminant analysis of FC, CRP and FBG shows a discriminant level of 80%. In this way, 74% of SD patients and 85% of S patients were correctly classified by the model.

### 3.4. Vitamin D Progression

**Figure 8:**
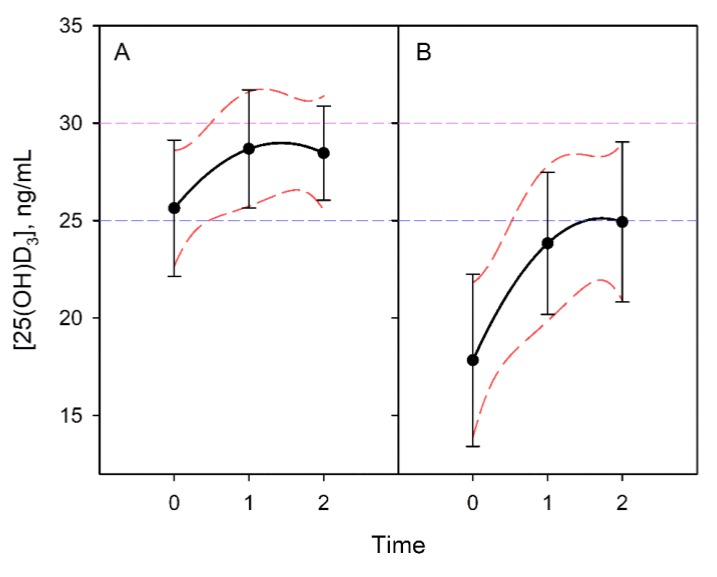
Evolution of vitamin D content in nonsupplemented individuals (**A**). The graph presents the lines of regression of the contents and their slopes. Active supplementation (**B**) did not guarantee levels of sufficiency in these patients.

The progression of vitamin D levels over time was evaluated. Patients were divided according to active supplementation. The mean vitamin D determination in the third event did not reach more than 25 ng/mL of vitamin D despite supposedly adequate supplementation. The Bonferroni test indicated significant differences between situations 0 with 1 and 0 with 2, while showing no differences between situations 1 and 2. This behaviour was the same in the group of patients who were supplemented and not supplemented (Figure 8).

### 3.5. Categorical Variables Study

**Figure 9:**
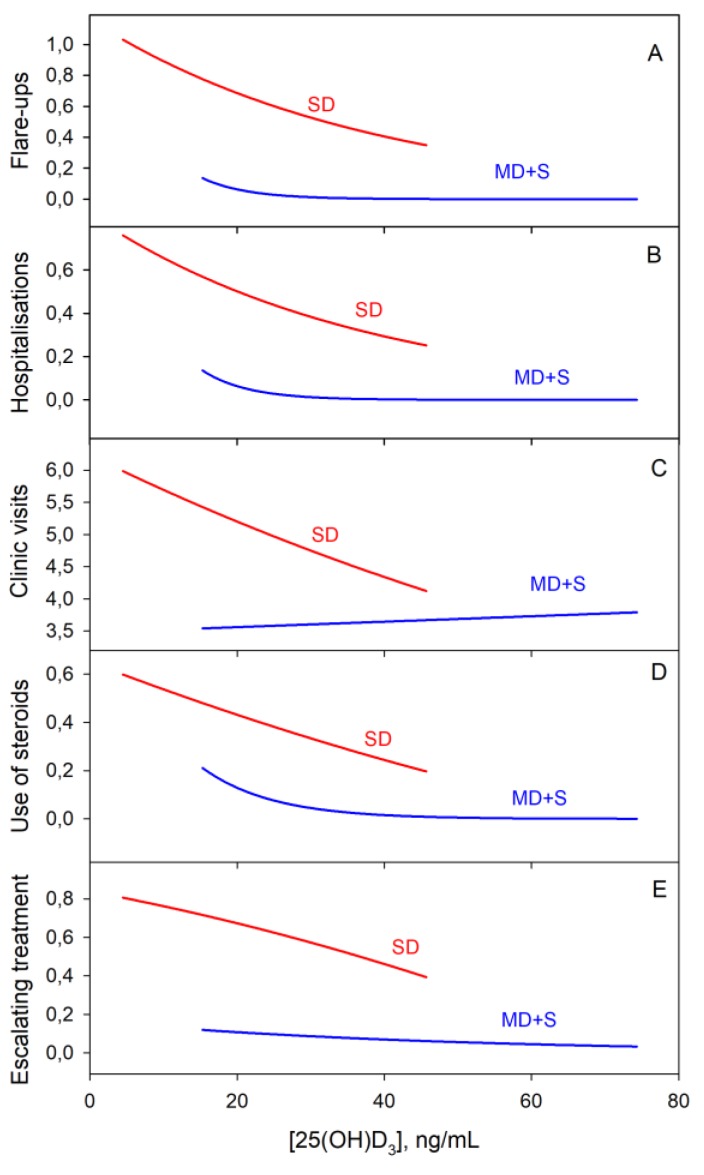
Logistic regression of clinical disease activity markers with vitamin D content. From A to E: Flare-ups, hospitalisations, clinic visits, use of steroids and escalating treatment, respectively.

For the categorical variables, a logistic regression analysis was used. Negative binomial regression showed an association between the SD group and a higher probability of having flare-ups, hospitalisations and clinic visit (*p*-value = 0.002). Graphically, it can be seen that for vitamin D levels below 20 ng/mL, the flare-up and hospitalisation rates increased exponentially regardless of the group (Figure 9). 

There was a correlation between those patients in the SD group and the probability of receiving treatment with steroids. Another correlation was also observed with the probability of introducing changes or the need for escalating treatment. All these results were statistically significant (*p* < 0.001).

## 4. Discussion

In this retrospective longitudinal study, we succeeded in describing an inverse correlation between serum vitamin D levels and both systemic inflammatory markers (CRP) and intestinal inflammatory markers (FC). Our findings confirm that vitamin D levels are also correlated with clinical disease activity, as represented in hospitalisations, flare-ups, number of clinic visits, use of steroids and changes in therapy. Additionally, we evaluated the vitamin D progression under oral supplementation, finding an insufficient absorptive capacity.

Other studies support the association between vitamin D and inflammation. In a cross-sectional study, Meckel et al. [32] demonstrated that serum 25(OH)D_3_ concentrations are inversely correlated with endoscopic and histologic inflammation and disease activity. In addition, they found that 25(OH)D_3_ concentrations correlated with the mucosal expression of VDR as well as epithelial junction proteins. Moreover, in a retrospective cohort of 60 patients, it was reported that vitamin D was inversely correlated with erythrocyte sedimentation rate (ESR), FC and clinical disease activity measured by Harvey–Bradshaw Index > 5 [36].

Supporting the results of our study, Kabbani et al. [37], in one of the largest prospective cohort studies, demonstrated that patients with low vitamin D levels required more hospitalisations, biologic therapy, steroids, surgeries and healthcare utilisation. We have demonstrated a strong correlation between vitamin D and FC as other authors [38] have done in similar studies. We describe an inverse correlation between vitamin D status and systemic inflammation (CRP levels) in UC. In another prospective study, Gubatan et al. [39] demonstrated that those patients with vitamin levels below 35 ng/mL were at greater risk of future clinical relapse. 

However, there are some uncertainties not resolved yet concerning vitamin D influence on IBD. Several translational studies are emerging to attempt to understand its hormonal pathophysiology, and there are some interventional studies trying to evaluate its active supplementation. Despite the importance of sunlight exposure in vitamin D levels, only a few studies have considered the season where the sample was collected. Baseline vitamin D levels are not yet established to evaluate deficiency and sufficiency. Thus, it is difficult to assess which patients are at real risk of developing clinical consequences. In our study, we set that limit at <15 ng/mL, but other authors set it at <20 or <35 ng/mL. The extent of the supplementation that should be given to our patients and what leads to sufficiency during IBD clinical activity is also unclear. 

In our study, several limitations must be considered. Despite describing a strong association between vitamin D deficiency and higher inflammatory markers and disease activity, we cannot confirm a causal relationship due to the retrospective collection of data. A notable cohort study of patients was included in our study, but the inclusion enrolment was in only one single centre by the main researcher. Thus, methodological flaws are possible, including selection bias. Finally, our study is observational and limited by the inability to account for potential unmeasured confounders. Although we evaluated active vitamin D supplementation, we did not consider baseline dietary vitamin D intake, summer or winter season measurement, body mass index, physical activity and adherence to medical therapy and supplementation. There is a relationship between vitamin D receptor genes (VDR), autophagy and gut microbial assemblage that is essential for maintaining intestinal homeostasis and contributes to the pathophysiology of IBD. However, genetic data regarding VDR polymorphisms has not been considered in this study.

Garg et al. [40] designed an interventional study on vitamin D supplementation where the enrolled IBD patients were given up to 10,000 IU (international units) of oral cholecalciferol. Despite achieving improved symptom-based activity scores and a mean of 41.6 ng/mL of 25(OH)D_3_, no significant reduction in FC levels was observed. It is remarkable that there was no control group in this study. Sharifi et al. [41], on the other hand, in a randomised trial, demonstrated a significant decrease in CRP and ESR levels with a single dose of 30,000 IU intramuscular vitamin D supplement. These results support the findings in our study in terms of insufficient supplementation. Doses recommended by guidelines, although suitable for bone health, appear to be clearly insufficient for IBD patients. Considering chronic oral malabsorption, future studies are warranted to explore significant differences in clinical scores or intestinal inflammation depending on the route of vitamin D administration. A higher target 25(OH)D_3_ concentrations during active supplementation should also be evaluated to clarify beneficial outcomes in IBD patients.

## 5. Conclusions

Vitamin D deficiency is now recognised as another predictor of clinical disease activity according to recent research. This study demonstrates that low serum circulating 25(OH)D_3_ is associated with intestinal inflammation (FC) in IBD and systemic inflammation (CRP) in UC. Deficiency of 25(OH)D_3_ is associated with more hospitalisations, flare-ups, use of steroids and escalating treatment. Standard vitamin D oral dose of supplementation is probably insufficient to reach normal serum 25(OH)D_3_. Vitamin D intervention studies are warranted to determine whether raising serum 25(OH)D_3_ level with higher doses of oral vitamin D in IBD may reduce intestinal inflammation or disease activity.

## Figures and Tables

**Table 1:** Statistical parameters obtained from vitamin D and inflammatory markers grouped according to vitamin D content.

				CI ^a^ (95%)				
Variable	Group	Mean	StDev	CI−	CI+	Skewness	Kurtosis	MSSD
Vitamin D	S	40.43	12.7	37.0	43.9	1.34	1.42	78
	MD	24.36	4.7	23.3	25.5	0.32	−0.16	17
	SD	17.83	8.1	16.3	19.3	0.85	0.39	65
								
CRP	S	2.28	2.37	1.8	2.8	1.82	3.5	1.5
	MD	2.80	3.07	2.3	3.3	1.71	3.01	4.3
	SD	23.6	51.0	16	31	4.48	24.2	1102
								
FC	S	54	64	39	69	4.09	19.72	2567
	MD	141	164	111	172	1.81	3.00	15,020
	SD	868	1278	665	1070	2.31	5.24	508,909
								
FBG	S	423	93	401	445	0.35	−0.51	4140
	MD	412	107	392	432	0.57	−0.13	6703
	SD	497	133	475	517	0.08	−0.77	5576

^a^ confidence interval; MSSD: mean of the squared successive differences.

**Table 2:** Robust statistical parameters obtained from vitamin D and inflammatory markers grouped according to vitamin D content.

							CI ^a^ (95%)	
Variable	Group	Min	Q1	Median	Q3	Max	CI−	CI+
Vitamin D	S	20.5	32.2	36.2	43.0	74.30	34.1	43.9
	MD	15.3	20.6	24.7	27.4	37.5	22.4	25.5
	SD	4.5	12.2	15.1	22.7	45.7	14.3	19.3
								
CRP	S	0.20	0.60	1.45	2.97	11.8	0.9	2.0
	MD	0.20	0.60	1.50	3.82	16.5	1.1	2.2
	SD	0.30	1.90	5.60	15.6	364	4.5	7
								
FC	S	19.6	26.7	29.7	50.7	434	28	37
	MD	17	27.6	63	229	738	43	98
	SD	19	97	346	1065	6450	216	444
								
FBG	S	262	356	411	491	664	387	446
	MD	201	325	406	475	700	365	432
	SD	151	390	478	602	813	456	502

^a^ confidence interval.

**Table 3:** Baseline clinical characteristics of patients meeting inclusion criteria.

Clinical Characteristic		Number of Participants (Percentage) or Mean ± Standard Deviation
Age, years		43.7 ± 15.9
Female sex		39 (46)
Crohn’s disease		60 (71)
Ulcerative colitis		25 (29)
Perianal disease		4 (5)
Extraintestinal manifestations		10 (12)
Vitamin D supplementation		44 (52)
Current therapy	Anti-TNFα	47 (55)
	Vedolizumab	8 (9)
	Ustekinumab	1 (1)
	Steroids	21 (25)
	Azathioprine	31 (36)
	Methotrexate	2 (2)

**Table 4:** Median and interquartile range of FC and CRP grouped by vitamin D levels for patients with and without vitamin D supplementation.

		FC, µg/g		CRP, mg/dL	
	Group	Median	Q3–Q1	Median	Q3–Q1
Not supplemented	S	33	25	1.3	1.8
	MD	85	211	1.9	4.2
	SD	524	2230	8.9	3.1
Supplemented	S	29	38	2.3	4.6
	MD	55	328	2.1	4.9
	SD	362	943	6.7	3.1
